# Perceptions and meanings of living with Parkinson’s disease: an account of caregivers lived experiences

**DOI:** 10.1080/17482631.2021.1967263

**Published:** 2021-08-20

**Authors:** Supreet Kaur Bhasin, Ishita U. Bharadwaj

**Affiliations:** Department of Psychology, University of Delhi, New Delhi, India

**Keywords:** Parkinson’s disease, caregivers, narratives, existential phenomenological analysis

## Abstract

**Purpose:** Current study looked into caregiving process of those dealing with family members suffering from Parkinson’s disease, within the changing social milieu in India. It aimed to understand the experiential and existential impact on the lifeworld of caregivers.

**Method:** Narrative interviews of 10 female caregivers referred by neurologists were gathered. Employing Existential Phenomenological Analysis, the caregiver experiences were understood phenomenologically within an existential framework, six themes were generated.

**Results:** Themes were – Becoming a caregiver: Undertaking immeasurable and unrelenting responsibilities; Rising patient-hood of one’s family member: pain of losing the person in the patient; Experience of altered temporality: living in pain with the uncertainty and duration of the disease; Encountering meaninglessness: dwindling faith in principles of life; Existing as a “Being For” and not “Being With”: a caregiver’s self-estrangement and blurring of Identity and lastly Self-Preservation through brief moments of respite: coping with caregiving.

**Conclusion:** The study illuminated how caregiving is experienced by an individual at a process and psychic level by shedding light on the conflicts, concerns and exhaustions endured by them. Adopting an existential approach in healthcare setups can aid in moving closer to felt experiences of these caregivers and in developing integrative and meaningful interventions for enhancing their well-being.

## Introduction

Parkinson’s disease (PD) is a multisystem neurodegenerative disorder associated with both motor (tremor, rigidity, bradykinesia, akinesia and balance impairment) as well as non-motor impairments such as depression, sleep disturbances and pain (National Institute of Neurological Disorders and Stroke, 2013; Sveinbjornsdottir, 2016). It is an age-related illness, and usually arises around the age of 50–65 years (Abbas et al., 2017). As per the WHO, Parkinson’s disorder is the second most prominent neurodegenerative disease (Pringsheim et al., 2014).

The range of symptoms in PD can constantly fluctuate on a daily or even hourly basis within the individual, giving rise to enhanced exhaustion and complications (Gunal et al., 2002; Haahr et al., 2011). Furthermore, for many people the medication for Parkinson’s disease is only effective for approximately 10 years (Rizek et al., 2016) and although techniques like Deep Brain Stimulation (DBS) have emerged as fruitful option, awareness and access of the same are still limited in a country like India (Haahr et al., 2018; Malek, 2019). Additionally, employing DBS also entails provision of psychosocial support to adjust to the remaining challenges of everyday life (Haahr et al., 2020). Consequently, many of the sufferers live with this disease for the rest of their lives and, as the disease advances, patients become more and more disabled, until eventually very little movement is possible (Bonner et al., 2020; Sunvisson, 2006). Following such progressive decay of functional abilities, the disorder renders an individual incapable of taking care of his/her own self, leading to complete dependency.

Parkinson’s disease thus affects the entire family and an extended community of friends and loved ones, since the burden of caring for the person with Parkinson’s disease is largely borne by her/his caregivers. In recent literature, a caregiver has been defined as an individual who helps with physical and psychological care for a person in need (Abendroth et al., 2012). With most of the caregivers being family members (known as informal caregivers), this role is usually donned by the spouses (61-70 years old in India, 75 years or older in USA), or adult children (AARP and National Alliance for Caregiving, 2020; Wandrekar et al., 2014) 2018). Since Parkinson’s disease is greater among men, the number of females enwrapped in the caregiving role are higher (Hirsch et al., 2016; Surathi et al., 2016). Consequently, more female than male caregivers are involved in daily care activities for the person with PD, in terms of assistive, supportive, and compensatory functions (Williams et al., 2016).

Caregivers of individuals suffering from PD perform a range of tasks such as aiding with activities of daily living, including bathing, toileting, dressing, transferring, cooking, eating, managing medications, making financial decisions etc. (Smith & Shaw, 2017). They are required to be available round the clock each day, constantly adapting themselves to the unpredictable nature of the disease and the demands of the patient. This often results in an experience of caregiver burden (Abendroth et al., 2012; Leiknes et al., 2015; Martinez-Martin et al., 2008; Ovallath, 2020).

Additionally, while informal caregiving, on one hand, could generate high role satisfaction, sense of accomplishment and emotional fulfilment in taking care of a loved family member (López et al., 2005), on the other hand, it enhances the possibility of caregivers facing health risks, emotional strain, and mental health problems (Mosley et al., 2017; Nene & Yadav, 2020; Roth et al., 2009). Research has shown that living with the degenerative nature of the disease often results in severe negative emotional impacts at the family level for caregivers (Tan et al., 2012). Caregiver experiences have highlighted feelings like – anxiety, frustration, constant worry, and loneliness, as they deal with the unexpected and profound physical and behavioural effects of PD on their family members (Smith & Shaw, 2017).

However, the caregiving beliefs, orientation, familiarization, and behaviours are vastly influenced by the cultural contexts (McDermott & Mendez-Luck, 2018). With an increase in the ageing population of India and subsequently rising cases of PD, a higher number of individuals, are having to assume the caregiver role, with most of the PD caregivers being 61–70 years old (Radhakrishnan & Goyal, 2018; Wandrekar et al., 2014). In India, the allocentric and collectivistic values and norms place greater emphasis on the well-being of the family over self (Kadoya & Khan, 2017). As a result, caregiving is believed to be a common part of the social and familial life of an individual. It therefore becomes essential to delve into challenges experienced by the caregivers, while embodying the caregiver identity based on their social and moral responsibility.

Vast research both globally and in India has focussed on the economic burden and quality of life of the caregivers (Greenwell et al., 2015; McLaughlin et al., 2011; Peters, 2014; Sanyal et al., 2015); however, less work has been done to understand the emotional, psychosocial, and experiential impact on the PD caregivers. Moreover, there is a huge paucity of explanatory models accounting for the subjective experiences of PD caregivers in the current socio-cultural milieu of India (Sanyal et al., 2015). Previous researches have also highlighted reaching towards the “insiderness” of the patient as valuable in facilitating meaningful and deeper engagement with the caregiving role (Todres et al., 2014). But to cognize the experience of life as a caregiver, it is essential to immerse in the lifeworld of the caregivers and gain an insider perspective from them (Smith & Shaw, 2017) (2017). How following a disruptive life event, the caregivers perceive their sense of belonging to the world and reposition themselves in the altered spatial and relational environment, needs closer examination.

Emerging from these concerns, the current study investigated the caregiving process of those dealing with family members suffering from PD, within the changing social milieu in India. It focused on developing a deeper understanding of the journey of their self-identity, their existential concerns, and navigation through the varied roles demanded of them.

## Method

The current study was carried out in Northern Delhi, during the period of August 2016-July 2017, by the first author during the course of her post-graduation.

### Research Design

The current study employed qualitative research design. In-depth personal narrative interviews were performed with 10 participants. In particular, a phenomenological inquiry was incorporated to capture the lived experience of caregiving for a family member.

This was because, phenomenological investigation aids in capturing the essence of an experience (Moustakas, 1994; Speziale & Carpenter, 2007) and has therefore been critical in family research (Dahl & Boss, 2005). Individual accounts were analysed using a theoretical framework of existentialism as put forth by Heidegger (1962)and Yalom (1980). Heidegger’s principle of existentialism perceives an individual to be an inseparable entity from the world he/she is embedded in. As a result, it enables the researcher to delve into the lifeworld of the being, and looks at the interrelationship between various elements of the lived reality of the individual. It does so by accounting for the spatiotemporal transitions and the bodyhood experiences encountered by an individual (Heideggar, 1962; Minkowski, 1933/1970; Van Manen, 1997). Additionally, existentialism grants, recognition of the presence of existential dimensions like loneliness, meaning, purpose, death and freedom, in the lifeworld of every being, while being sensitive to the dynamic nature of these dimensions, with some rising into dominance depending upon the situation and context (Gadamer, 1975/1989; Yalom, 1980). Finally, existentialism as an approach emphasizes the construction of self by focusing on issues of existence and co-existence (Yalom, 1980), which becomes critical in understanding the everyday experiences of family caregivers, co-habiting with the ailed.

### Sampling Procedure and Participants

The research included the participation of 10 women caring for their family members with PD. Caregivers of patients with PD, seeking treatment from two central Delhi hospitals- one government and one private centre with specialized departments catering to people with Neurodegenerative Disorders were included. Information about the research study was circulated through word of mouth as well as by posting flyers with details of inclusion and exclusion criteria within the respective neurology departments of the two hospitals. Patients were referred by the neurologists working at these hospitals. The purpose of the study was explained to the referred patients and subsequently potential participants serving as caregivers to the patients, were identified. Participants provided with their consent were enrolled in the study. The circumstances of the caregiving situation varied, in terms of the relational matrix, comprising spouses, daughters and daughters-in-law functioning in the capacity of the caregiver. The nature of sampling was purposeful.

Inclusion criteria for the participants were – serving in the capacity of a caregiver to a family member with PD for at least a year, any position in the relationship matrix, able to communicate verbally in English or Hindi. Following the explanation of the research study, written informed consent was obtained from the participants. Rapport was established with the participants before the commencement of the narrative inquiry, and adherence to all ethical guidelines was ensured. The narrative interviews were held at the residence of the caregivers since it allowed them to take care of their responsibilities while participating in the inquiry.

### Data Collection

The tool of inquiry was narrative interviewing. The reason for choosing narrative interviewing as a method of data collection was because narratives are constructive in nature, and they facilitate an individual in pulling together different parts of their self, to represent the subjective experience of existing in the world, as well as to share the inner representation of their self-other relationships (Michael, 2010; Ricoeur, 2002) As the caregivers absorbed themselves into the world of the patients, narratives provided them an opportunity to voice the disruption and transitions encountered by them, while repositioning themselves as per the needs of the patients.

Individual in-depth narrative interviews, borrowing from the frameworks of Wengraf (2001) and Anderson and Kirkpatrick (2016) were conducted. This involved encouraging the participants to share their life events in a story-like manner, such that they were linked in time and meaning, both. These were audio-recorded and transcribed. The interviews were carried out face-to-face, at the respondents’ home.

Open-ended questions based on rigorous literature review and in-depth discussions with the first author’s mentor were designed. These questions centred around the journey of becoming a caregiver, the challenges encountered during the course of it and the experience of living as a Parkinson’s Disease caregiver. A few of these were: “If you were to tell me about your life story as an individual co-habiting with a Parkinson’s disease patient, what would you like to share?”; “If you were to describe how has donning the role of a caregiver changed your life?”; “Tell me about a typical day, living with PD.” Such narrative expressions aided in understanding the “inner” and “outer” worlds of the individual, and their system of relevancy in the context of their caregiving experience.

### Data Analysis

Existential Phenomenology aims at understanding the experience of being in the world by an individual, while accounting for her/his situatedness. It focuses on encapsulating the essential structures guiding an individual’s ability to interact with and find coherence in the world and understanding the reflective mechanisms aiding in doing the same (Von Eckartsberg, 1971, 1986; Yalom, 1980). By focusing on the sense of belonging of an individual, it enables one to understand the fluidity of an individual’s experiences across time, their perceptual shifts, while engaged in meaning making of their life, their array of emotions and their extension of self for the other (Bogard, 2010; Vaughan & Kluger, 2018). As these participants embodied the caregiver role, an understanding of changeovers in their lived bodily experiences, their sense of loss and unfamiliarity in the everydayness of life, and the alterations in their relationship with self, the other (patient) and consequent sense of grounding in the world, could be best illuminated by existential phenomenological approach.

The data were thus, interpreted using the principles of existentialism (Heideggar, 1962; Yalom, 1980) in conjunction with existential phenomenology as a method of analysis, as proposed by Polkinghorne (1989). It involved identifying the meaning units in a narrative and looking at them through the perspective of the participant while accounting for his or her subjectivity and context. Thereafter, the text was re-read and meaningful units of analysis that best captured the essential qualities of that interview were selected. It involved observing patterns of existential conflicts, language use, past-future comparisons, metaphors/imagery, and investigating the critical concerns faced by the caregivers. The third stage involved attempting to provide an overall structure to the analysis by grouping the identified meaning units of analysis into “clusters” or concepts. This was done to arrive at a group of themes and to identify super-ordinate categories by looking for possible connections between the meaning units of analysis, following which these themes were organized in a table. During the entire process, notes of any thoughts and reflections of the researcher were also maintained to account for her subjectivity and positionality.

However, the method adopted by EPA is a cyclical process, so the researcher proceeded through several iterative stages to develop a close description of learning to live as a caregiver of PD.

## Results

The participants were ten late-middle-aged females, ranging from 45 to 70 years of age and were currently residing in the national capital, Delhi. All the participants belonged to middle to upper class socioeconomic backgrounds. Their narrative interviews ranged from 90 to 120 minutes. Six of the caregivers were wives of the patient, two were daughters-in-law and one of them being a daughter to her ailed father. The years of involvement with the caregiving experience ranged from a minimum number of 5 years to a maximum of 18 years. Based on the analysis, six themes were identified (see Table I for summary)

**Table I.** Themes generated and the corresponding clustered meaning units of analysis

Clustered Meaning Units of Analysis	Subordinate Theme
Managing “Self-management needs” of the “Other”	Becoming a caregiver: Undertaking immeasurable and unrelenting responsibilities
Adapting to sudden shift in power dynamics	
Newer roles compensating for his absence	
Moral reasons and dutifulness	
Relational choicelessness in caregiving	
Moral dilemmas over quality of care	Rising patient-hood of one’s family member: Pain of losing the person in the patient
The familiar person becoming unfamiliar patient	
Lack of companionship	
Shadowing the time zone of the care-receiver	Experience of altered temporality: living in pain with the uncertainty and duration of the disease
Fragmented sense of time and body	
The disease transforming into endless, repetitive routine	
Close witness to the suffrage of a loved one	Encountering meaninglessness: Dwindling faith in principles of life
Confronting the existential truth: absence of cosmic meanings	
Disenchantment from the virtuous path of life	
Rising mechanical engagement with the patient	Existing as a “Being For” and not “Being With: A caregiver’s self-estrangement and blurring of Identity
Weighed down by Compulsion: experience of constant moral heaviness	
Reduced to only a caregiver: Perception of self and other	
Neglecting one’s own well-being: self-sacrifice	
The Non-legitimized patient-hood in the Caregiver	
Acceptance	Self-Preservation through brief moments of respite: Coping with caregiving
Family support	
Optimism	
Cosmic meaning to live	
Recreational distractions	

## Becoming a caregiver: undertaking immeasurable and unrelenting responsibilities

This theme focuses on additional demands silently borne by the caregivers while embracing their caregiving role. It sheds light on how with the increasing functional disability of their ailed family members, their responsibilities increased exponentially.

The caregivers found themselves engaged in constant care, from performing additional tasks of managing the patient’s symptoms to aiding in his self-management needs. However, most of the participants also confessed experiencing a sense of lack of choice and discomfort while doing the same. They highlighted, with reluctance that as a female caregiver it was imperative for them to fulfil these uncomfortable needs of the patient, and while doing so, also tried to preserve their self-identity. For instance, Sunita (Pseudo-names used to ensure confidentiality), an old-age participant, shared her disgust in having to clean her husband’s soiled clothes as well as his dump, while acknowledging the lack of any other option available to her:

I have also cleaned his faeces … what other option do I have, if I- his wife won’t do it who else will, I have to do it … .so I am doing it, although I hate doing it … I find it extremely hard and … to be honest even repulsive, but as a wife, I’ll have to do it … there is no other way … so I do it!!

In addition, the cognitive decline of some of the patients added to the burden of the caregivers. They had to acquire new knowledge, deal with greater degree of uncertainty and immerse themselves in various unfamiliar undertakings.

Nirmala shared how she was put in the difficult situation of handling finances, an arena which for 47 years had been handled by her husband exclusively:

Similarly, Savitri shared that due to deterioration in her husband’s health, there was a sudden surge of workload:

He used to perform various tasks of the home, but now with his trembling limbs, his movement has become vastly limited, so apart from the household chores, there are several other things that now I need to account for.

Apart from having to acquire new skills at an old age, some of the participants were compelled to single-handedly bear the onus of taking all the critical family decisions. This sudden shift to be a leader from either being a partner or a follower in the decision-making process was found to be burdening and difficult to manage.

Thus, as the caregivers loaded themselves with innumerable tasks and responsibilities, they also had to accustom themselves to the changing power dynamics and bear with a sense of vulnerability, indubitably hoping for the next day to be better than the current one.

## Rising patient-hood of one’s family member: pain of losing the person in the patient

This theme highlights the pain and moral dilemma experienced by the caregivers as the disease took over their family members, and with increasing time he became an unfamiliar identity to them. They were coerced to unwillingly give up on shared meaningful activities, some of them experienced lack of companionship and heightened loneliness.

For instance, Dimple, shared how in day-to-day routine she missed her husband’s mental presence with her at any given point of time:

I am with him physically and mentally even when I am drained out because otherwise, I cannot take care of him, but I find that he is mentally absent sometimes, mostly because of his preoccupation with his illness and the diminishing physical and mental faculties … that in itself is upsetting, because he used to look after me, he took care of everything for me

Likewise, others felt that the disease brought uncertainty not just in terms of physical and mental challenges, but also in terms of the emotional and relational connectedness with their family members. This also emerged because the constrained mobility of the patient, intertwined with different dimensions, which gave meaning to the caregiver’s lifeworld. For instance, Kusum, shared how her husband’s illness manifested as his inability to follow an active and disciplined routine like before. This resulted in her having to not only bear the loss of her partner in shared meaningful activities of everyday life, but also having to anticipate and act in accordance with his possible deteriorations in near future.

It was such narratives, which gave an insight into the emotional turmoil experienced by the caregivers as they battled with their inner void and desperate search for the “person” in their patient.

As the familiar spaces and known grounds of relationships, participation and interaction distorted for the participants, some of them also confessed to becoming overly tied up in the symptom management of their family members. As a result, a part of them felt driven to reduce a person’s saliency only to his illness. This generated within them additional angst and discontent towards their own selves.

Kaveri, caring for her father-in-law, revealed experiencing immense anxiety and guilt for tying him up whenever he became difficult to manage. It seemed to her as if she was treating him no different from an object:

I have to tie him up … earlier when doctors suggested that and I had to, it used to impact me, after all he is my father in law … .but often he becomes too difficult to manage, he tries to get up and run but falls, gets aggressive, It just becomes too much to handle, so I find no other way but to tie him … .now it is very mechanical for me, but sometimes it just makes me question myself, I feel ashamed that I resort to such extremes … .now it’s a different kind of pain … a pain of disappointment and exhaustion!!

Varying from such candid to subtle expressions, the dissonance felt due to the dichotomy between at one end being a caregiver and lending beneficence and on the other hand becoming oblivious to the person in the patient, became evident.

## Experience of altered temporality: living in pain with the uncertainty and duration of the disease

Experience of time was one of the most salient themes for the informal caregiver since it was intertwined with their bodily sense of being as well.

Most caregivers described their time in the caregiver role as unending and extremely time-consuming. Sunita and Gargi often used phrases like “a sense of always being on call” and “there are no off days from caregiving” while sharing their experiences, indicating the need to be vigilant, working with a sense of urgency, and expecting the unexpected.

This also arose since the disease brought about a change in the concept of time for the care receiver as well. Shelly’s narrative highlighted this element of life:

He is often restless and has difficulty sleeping at night because of certain challenge with his eye muscles, so he often calls out to us at night. So, I need to repeatedly get up and cater to him … it gets quite draining, I often lose track of night and day. Sometimes I don’t even get any sleep and have to continue with my remaining responsibilities for the rest of the day … .so you know it feels as if time is just stretching into eternity and there is no rest period!!

For other caregivers, like those who were working additional jobs, a similar experience resulted in the need for more time.

Another tangent of fragmented sense of time originated from the inability of a caregiver to move on and have a definite schedule to lend a sense of coherence to their days. Corresponding to the narrative of Dimple, others like Nirmala, Sunita and Kusum also shared, how their entire day was spent hovering around the PD patients:

Since his illness, the entire discipline of the house has gone for a toss … … everything is centred around his needs and his timeline.

These narratives emphasized how their partner’s gradual decline impinged on the caregiver’s life, creating a loss of autonomy and lack of any opportunity for them to relax.

In some instances, the same routine was often required to be repeated day in and day out. It brought within the caregivers, a sense of monotony and disengagement with the patient and his needs. Sakshi, for instance, shared feeling trapped in a never-ending loop when her father time and again called out to her for hours at a stretch for the tiniest of things. This spawned a sense of distance and mechanized engagement with her father, furthering a sense of guilt.

Initially I used to try and soothe him out or reason with him, but now I am just too tired to explain anything, I feel sad for not being more patient, but I can’t ….

Thus, caregivers were often inevitably caught in the care receiver’s sense of time. The time and effort involved in caregiving, also took a physical toll, engendering the experience of a fragmented sense of body among the caregivers. The narratives reiterated the state of constant exhaustion, a change in the overall structure of world experience and their outlook towards one’s future.

Savitri, caring for her husband, echoed these concerns:

My life has changed to a full 360 degree!! I have lost weight, I have lost my hair, I have lost my strength and at one time I had lost my appetite as well … I have lost my sense of well-being … I was initially very fond of dressing up, how I look, my presentation, my food … but this is so physically draining that I do not bother much now, I do not have the energy to look at all this, nor do I get any sense of pleasure from anything at all

The roles of caregiver is a long one, not limited to a particular point in time but rather drawn out over months or years, as a result, it was common that a sense of hypervigilance and insecurity prevailed due to the uncertainty surrounding the caregiver role.

## Encountering meaninglessness: dwindling faith in principles of life

This theme refers to the disenchantment of the participants in the cosmic patterns and meaning systems. It reflects their disheartenment at witnessing a virtuous person suffer choicelessly from an unrelenting progressive disease.

Gargi expressed her perturbation at discovering the meaninglessness of the idealized virtues, and the imbalanced equation of altruism as a fact of life. She shared:

He has been one of the kindest people I have ever met, he goes out of his way to make people feel better … he has never hurt a soul. Then why him? Why would such a terrible problem emerge for such a good soul … .it doesn’t make sense ….

The narratives of the individuals positioned as caregivers depicted how they also experienced an existential vacuum by virtue of being an observer and a living partner to the deteriorating status of their family members. These shades of existential vacuum ranged from agitation, mistrust, meaninglessness, and unending questions towards the future. Shelly shared how witnessing the suffrage of a good person was an emotionally heart wrenching part of caregiving. She found herself struggling with feelings of betrayal towards God, uncontrollable frustration, and a sense of pointlessness towards life:

He has been an extremely helpful person throughout his life … you know we have always been told that good things happen to good people, then why would God make such an innocent and good-natured man suffer like this?! Sometimes I feel angry at God for putting him through this. What’s the point of honouring these belief systems, when despite your goodness you can experience your life rotting away!! I have completely lost my trust in any of the systems governing our life and that hurts … I don’t know now what to believe in and what not to … it is scary to imagine what lies ahead ….

## Existing as a “Being For” and not “Being With: a caregiver’s self-estrangement and blurring of identity

This theme looks at the experience of estrangement of caregivers with their very own being. It draws attention to how the process of embodying the caregiver role, culminated into alienation from the central core of one’s individuality, spawned challenges of spatiality and manifested in the form of sense of patient hood within them. Most of the participants inevitably shed light on the experience of being confined, feeling consumed, insignificant, and invalid.

One of the participants shared that she was often confined to time or space, since in her absence, the care recipient felt a sense of dislocation and it was expected of her to conform without any complaints:

This is a major concern; I can’t go out at all … he needs constant care. Even if I step outside for a few minutes with my phone, he doesn’t like it … he feels with my absence he cannot function. I am happy to be there for him, after all I am his wife … but at times I also feel all alone because of that … .it is as if my concerns are unimportant … over the period of time it has made me feel that I am just a caregiver, I need not be heard! (Shanti)

It was not unusual for these female caregivers to be self-sacrificial to the extent of being completely disregardful of their own needs and desires. They often compromised on their health and social interactions, despite feeling a necessity for both, because of the responsibilities shouldered by them—both in terms of physical and moral accountabilities.

I am fond of eating, but for my health it is important that I walk as well. He doesn’t let me step out … so where do I walk, within this tiny space? I compromise for him and don’t go out. But there are times, when I feel that my needs and desires are discarded by everyone and even, I have been accustomed to do this for years … this bothers me at times. (Sunita)

Every Sunday we used to have relatives over or a meet up, but since almost a year and a half I haven’t been able to be a part of such gathering., We can’t host now, people feel they do not want to trouble him, and I can’t leave him alone … it somehow makes me feel really low and excluded … I used to be a very jovial person, but now I remain silent mostly, emotionally broken and extremely lonely. (Kusum)

For most of the participants, living as a caregiver meant enduring the burden of performance of one’s duties at the cost of self-neglect. Doing so, often required them to suppress their emotions and discount those characteristics of their being which provided them with wholeness. The caregivers were so absorbed in their role that they ceased to exist as separate individuals and instead expanded themselves to be taken over in entirety by the patient’s life choices. This depersonalization of oneself and the perpetual invisibility of self by oneself and others resulted in a need for validation, invariably giving rise to a patient-identity within them. This came to the surface when participants shared statements like:

I don’t know if I have anything left to say … .my life has been an expansion of his for years now … I have been so absorbed in my duties towards him, that I don’t remember myself as anyone other than a caregiver … . it’s been so many years now … I don’t feel much, just a sense of emptiness and sort of machine like … (Dimple)

Sometimes I feel that while serving him and looking after him, somewhere even I have developed this disease … now even I feel that my hands tremble at times and I have difficulty sleeping …. (Sunita)

Hence, the journey of caregiving manifested as a journey of self-estrangement for these individuals as well. Consequently, social exclusion, incessant loneliness, losing essential parts of one’s being and bearing with the pain of invisibility, became the defining elements of life as a PD caregiver. An escape from this plight was only possible if the identity of patient-hood devoured them as well.

## Self-preservation through brief moments of respite: coping with caregiving

This theme focuses on the mechanisms chosen by the caregivers to preserve their self-identity and seek some moments of respite from the unending role of caregiving.

Some of the participants found respite in their family members and diffusing responsibilities among them. Sakshi often took her daughters’ help, while Kaveri mentioned having an understanding partner aided in navigating through the exhaustive process of caregiving.

Although the participants agreed not being able to fully relax and experience a form of renewal or change, some of them attempted to step out of the physical and embodied spaces of caregiving by engaging in other activities of distraction for brief moments of time:

I usually don’t get much time for myself, but when he sleeps during the day, I try to plug in the radio on a low volume and listen to old soft songs … it helps me revisit the time when we went out and our children lived with us … that’s my small escape. (Savitri)

Participants also mentioned how conversations about events and affairs unrelated to the disease or their family members, aided them to experience normalcy. Some preferred reading books to find an escape in fictional narratives, while others engaged in looking at the positive part of their experience, to rationalize their circumstances and find ground.

However, an overarching mechanism which most of the participants engaged in was of finding meaning and acceptance by resigning to spirituality and fate.

I feel God is the answer to everything. Despite all ups and downs, if you ask for God’s help, if you ask for strength, he blesses you as well … My faith makes me believe that if God has added this element to our life, it would be for a reason and if we accept it and surrender to him, things will get better … sooner or later … but they will.

Hence, spirituality played a key factor in accepting the disease as well as the role of a caretaker for the participants.

## Discussion

This research attempted an in-depth exploration of the experience of living as a caregiver to a family member with Parkinson’s Disease. By seeking to understand the perception, means of sense making and the process of embodying of the caregiver role, it illustrated the existential impact of living with the disease, from the caregiver’s perspective. In congruence with previous researches (Abendroth et al., 2012; Buse & Twigg, 2018; Sullivan & Miller, 2015), it was found that as the participants transformed from being just a family member to becoming a caregiver, the demands thrust upon them and the responsibilities shouldered by them multiplied. However, accompanying these demands was an implicit commitment of bearing with unending uncertainty, a restricted sense of autonomy and a silent acquiescence of being self-sacrificial. This might be because, the social grand narrative, especially in our country venerates the selfless, compromising, and devoted woman. Thereby, as the women of the house, adhering to their moral obligations and abiding by the virtues of their social role, was critical for the participants (Kleinman, 2013; Navarta‐Sánchez et al., 2017). The burden of these moral obligations not only pushed the caregivers to continue exhausting themselves beyond their capacities, but it also spawned an additional struggle of constant moral dilemmas and emotional repression. As the caregivers tried to support their ailed family members, they had to submerge themselves in being the ideal facilitator for the patient, adopt new roles and persist in their role performance. This they did even in cases where they felt aversive towards it, while being mindful and sensitive of avoiding injury to the patient’s psychological faculties and sense of competence. This was extremely overwhelming for the caregivers, and often manifested in the form of emotional wear-out and feeling misplaced as was also found by Tan et al. (Kontrimiene et al., 2021; Tan et al., 2012). In addition, in moments of incapacitation, wherein the caregivers could not be thoughtfully involved with patient, they felt guilty for not being good enough (Viwattanakulvanid et al., 2014). This might be because, as highlighted by Jaspers (1971), the existence of an “I” germinates from interaction, engagement, and co-construction with each other. As the caregivers found themselves struggling to be sensitive towards the patient, and found their involvement to be mechanical, it was perceived as a reflection of the diminishing humanity within, and therefore upsurged a sense of anguish and worthlessness.

The caregiving journey was emotionally tormenting also because, while compensating for the absence of their family members in functionality, they had to endure unwarranted loneliness. In congruence with earlier studies with an ever-increasing threat of losing a sense of familiarity with their family members, the caregivers experienced heightened insecurity and chaos (Dunk et al., 2017). It gave rise to the experience of unrelenting void and an ontological sense of insecurity towards one’s position in the family, future-outlook, and one’s own well-being. With the progression of the disease, the motor and cognitive abilities of the patients declined and as supported by previous literature, (Boersma et al., 2017; Den Oudsten et al., 2011) the quality of relationship between the caregivers and patients was severely impacted. The negative mutuality of reciprocation, exhaustive emotional involvement and the 24 × 7 commitment to the patients, furthered the feelings of loneliness, by excluding them from the otherwise available social support systems (Bhimani, 2014; Haahr et al., 2012; Martin et al., 2015). Simultaneously, for some of the caregivers it also laid the foundations for existential guilt and existential loneliness. This was because, as the caregivers drove themselves relentlessly into the service of the patients, not always out of choice, it often weighed them down, withholding them from participating in their lifeworld with their fullest potential; thereby, as proposed by Heideggar (1962) engendering in them the existential guilt for inauthenticity. Furthermore, the countless caregiving responsibilities and adjustments that were required at their end, also created an altered perception of time for these individuals. In order to support their family members, the autonomy, routine and pace of life established by the caregivers over the years, was largely ruptured, as found in corresponding literature as well (Barken, 2014; Wright et al., 2015). As a result, the caregivers oscillated between never having enough time on their hands, to experiencing life as halted, repetitive, monotonous, and full of uncertainty. These frequent fluctuations and repeated attempts to assimilate their family members’ illness into their everyday life were inevitably tiresome for these participants.

In addition, as emphasized by existentialists (Heidegger, 1960; Yalom, 1980), every individual exists in this world on the foundation of the existential quality of relating and sharing oneself with others. By the virtue of co-existence, the caregivers related and personalized the experiences of the patient towards their own interpretation of the lifeworld. Consequently, one of the biggest struggles experienced was that of meaninglessness and powerlessness. Most of the caregivers openly expressed fear of uncertainty about their family member’s well-being, uncertainty towards their own socio-emotional positionality and towards their carer role, as has been observed in previous studies as well (Hurt et al., 2017; McLaughlin et al., 2011; Tan et al., 2012; Theed et al., 2017). This majorly arose because, as proposed by Yalom (1980), every individual possesses a sense of coherence and purpose in life and connotes the greater meaning of existence to cosmic meaning (Yalom, 1980). He proposed that human beings are comforted by the belief that there is some super ordinate and coherent pattern to life. However, with the advent of the disease, and the plight of witnessing the gradual deterioration of a loved one, their faith in the cosmic principles such as—the just world hypothesis and Karmic cycle was shattered, creating a sense of purposelessness and loss of meaning.

In this process, as the participants battled with uncertainty, existential guilt, loneliness and meaninglessness while accommodating the numerous commitments and responsibilities of the patients, they invariably associated excessively with their socially sanctioned identity of being the “ideal caregiver”. However, as proposed by Heideggar (1962) and Yalom (1980), when a person addresses oneself and acts in accordance with only what “they”- others expect of him/her, the individual succumbs to viewing oneself as no more than an intersectionality of one’s roles, social affiliations and desired characteristics. In the case of the caregivers, as they accepted being no more than a communal being, they overly associated with their caregiver identity and started to exist as a “being-for-the-other” and not as “being-with-the-other”. This self-sacrificial communal part of self often manifested as self–abnegation within themselves – with a neglect towards one’s sleep, physical health, emotional and mental well-being, as also found in complimentary research (Kartika et al., 2015; Sullivan & Miller, 2015). Furthermore, being acknowledged by their significant others as “only a caregiver”, feelings of rejection, disregard and isolation increased among the caregivers, as highlighted in similar researches (Bramboeck et al., 2020; Erlingsson et al., 2012; Greenwell et al., 2015; McRae et al., 2009). Consequently, surfacing out of the need to be visible as an individual in themselves, an inconspicuous need for and association with the identity of patient-hood emerged in some of the participants.

Furthermore, to overcome these existential conflicts, it became critical for the caregivers to align themselves to the life circumstances of the patient. At one end, the caregivers pushed themselves to continue being resilient and hopeful, on behalf of their patients, as iterated in the previous literature (Haahr et al., 2012). At the other end, to establish a common ground of mutually shared spaces, the caregivers extended their lived body into spatial dimensions of the patient, attempting to come together by physical as well as existential means. However, for them, spatiality also signified the ability to move between closeness versus distance from their caregiver role. Therefore, to seek some respite, for brief moments, they tried to psychologically remove themselves from the caregiving situation. For this purpose, they sought support in social interactions and conversations, recreational activities like book reading or listening to radio. This is in congruence with prior research, which has majorly highlighted the significant role of conversations and social support as key mechanisms to cope with the caregiver stress and preserve their self-identity (Abendroth et al., 2012; Lee et al., 2019; Smith & Shaw, 2017). However, as proposed by Yalom (2000), in the face of uncertainty, faith and meaning in the greater scheme of things, often acts as a support mechanism. This is more dominant in Asian countries like India, where faithfulness to God’s will and trust in the predetermined destiny form the basic principle of leading one’s life (Elsner et al., 2012; Kazemi et al., 2021). Consequently, resembling earlier research (Dekawaty et al., 2019; Elsner et al., 2012; Kartika et al., 2015), most of the participants of the current study also found coherence in their life and the strength to cope, by developing acceptance and submitting to fate and greater cosmic meaning. This not only provided them a mechanism to deal with the emotional distress, but also enabled them to develop a positive outlook towards their life and persevere towards their future with more meaning and hope.

## Conclusion

The current study focused on investigating the caregiver’s accounts and perspective of living with Parkinson’s disease. Taking this idiographic approach requires making sense of their subjective experience within their relational realm, and their sense of connectedness to various aspects that give meaning to life.

In the study the challenges salient to the caregiving experience were the additional responsibilities required to be shouldered by them, and the sacrifices made at the expense of oneself. In addition, difficulty in dealing with uncertainty, timelessness and an incessant progression of the disease was one of the prominent precursors identified, leading to burnout. The existential lens illuminated the anguish experienced by the caregivers while navigating the overarching disease ambiguity and consequent perceived meaninglessness. It also helped bring to light the isolation and emptiness experienced by these individuals as they ineffectively attempted to reconcile the past and present, while frantically searching for the familiar person in the patient. Battling with the persistent feeling of dissonance, they also had to adjust to the new-found reality of shifting meanings of time. To overcome these existential dilemmas and experiential challenges, the caregivers attempted to reposition and re-orient themselves towards their life by practicing self-preservation. They often relied on finding purpose in their caregiving role by surrendering themselves to greater cosmic patterns and spiritual meanings.

In conclusion, taking an existential phenomenological approach, the research helped in moving closer to the process of caregiving as experienced by the individuals, while accounting for their cultural, relational, and existential systems of operations. It gave an understanding of caregiving at a process level as well as psychic level for the individuals.

## Limitation and Future Implications

The limitations associated with exploratory research apply, that it is oriented towards simply gaining an experience and understanding of the phenomenon under study. The data in the present study were obtained from participants inhabiting urban and metropolitan scenarios from the upper middle class, seeking treatment at the central hospitals of the capital. Thus, the findings should not be taken to be a representative picture of the entire population of people identifying as caregivers to Parkinson’s Disease. However, since these findings bring to the forefront, the caregivers’ narratives of living with PD, embodying their interminable role, and enduring the brunt of this identity as an existential being, they can be valuable in developing an intervention model for such caregivers. These interventions, modeled by touching upon the emotional anguish, struggles of uncertainty, self-estrangement etc. can aid in providing a comprehensive grounding as well as upliftment to the caregivers, while accounting for the socio-cultural context of their reality. Furthermore, more research, examining the experience of the caregivers along with those of the patients can help in holistically understanding how living as a family unit specifically in collectivistic culture, while battling with Parkinson’s disease is felt.
